# Scutellaria baicalensis extract alleviates salpingitis and enhances egg production in laying hens via suppression of the NF-κB signaling pathway

**DOI:** 10.1016/j.psj.2025.106018

**Published:** 2025-10-23

**Authors:** Jing Zhang, Qianmei Zhang, Shiyong Wang, Yuechun Yang, Lingyan Zhang, Lihong Chen, Zhenglu Xie

**Affiliations:** a Institute of Laboratory Animal Science, Guizhou University of Traditional Chinese Medicine, Guiyang City, Guizhou Province 550025, PR China; b Jinshan college of Fujian Agriculture and Forestry University, Fuzhou city, Fujian Province 350002, PR China

**Keywords:** Scutellaria baicalensis, Laying hen salpingitis, Egg production, NF-κB, Antibiotic alternative

## Abstract

Scutellaria baicalensis (SB), a traditional Chinese herb known for its bioactive properties, was evaluated for its potential to mitigate salpingitis in laying hens. This study investigated the effect of SB extract (SBE) on salpingitis and explored its underlying mechanisms of action. *In vitro*, an inflammatory response was induced in HD11 macrophage-like cells using lipopolysaccharide (LPS), followed by treatment with SBE. For the *in vivo* trial, salpingitis was induced in laying hens via intraperitoneal injection of *Escherichia coli* and *Staphylococcus aureus*. Hens were subsequently treated with SBE or amoxicillin over a 21-day period. On day 21 post-infection, hens were humanely euthanized for sample collection. Analytical methods included antimicrobial susceptibility testing, histopathological examination, ELISA, quantitative PCR, and Western blotting. SBE exhibited no cytotoxic effects and ameliorated LPS-induced cytotoxicity in HD11 cells. *In vitro*, SBE modulated the expression of both pro- and anti-inflammatory cytokines. *In vivo*, SBE administration significantly improved egg production, with an average yield of 27-32 eggs per hen over 21 days compared to an average of 14 eggs in the infected untreated group. Plasma levels of IL-1β, IL-6, TNF-α, and IFN-γ were significantly reduced, while IL-2 levels were increased. Differential white blood cell counts (neutrophils, eosinophils, and lymphocytes) were restored to levels comparable to those in healthy birds. Gross anatomical and histopathological evaluations revealed reduced oviduct lesions and diminished inflammatory infiltration. Western blot analysis indicated that SBE suppressed NF-κB nuclear translocation from the cytoplasm to the nucleus through enhanced phosphorylation of AKT and IκBα, thereby inhibiting inflammatory signaling. This finding was consistent with in vitro results. Additionally, SBE exhibited direct antibacterial activity against relevant pathogens. These results indicate that SBE effectively alleviates salpingitis in laying hens by inhibiting NF-κB nuclear translocation via IκBα/AKT activation and concurrently improving egg production. The study supports SBE as a promising antibiotic-free alternative for managing salpingitis in poultry.

## Graphical abstract

**Figure:**
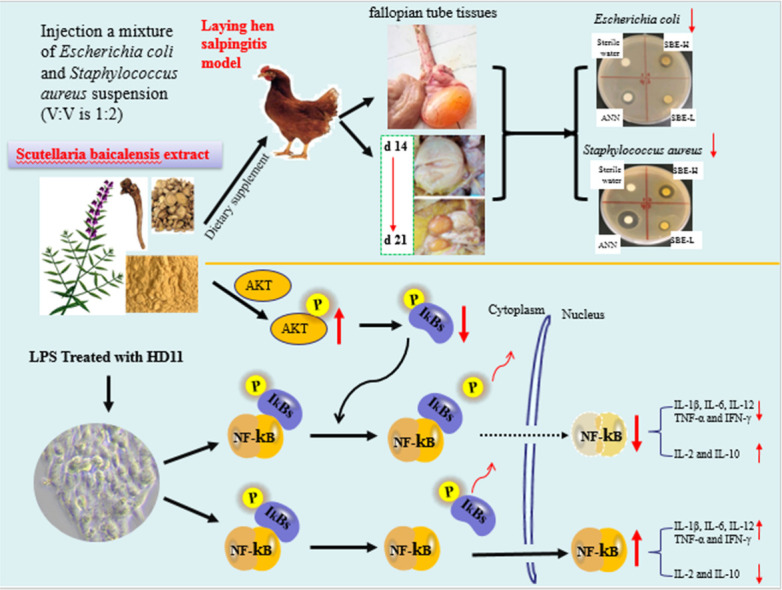

## Introduction

Salpingitis is a common and economically significant disorder in laying hens, particularly during peak production. It often leads to reduced egg production, impaired egg quality, and eggshell contamination, such as the presence of blood stains(Li et al., 2023; Liu et al., 2024; Saraiva et al., 2021; Wang et al., 2020; Yan et al., 2023). The condition is frequently associated with bacterial infections, primarily *Escherichia coli, Staphylococcus aureus*, and *Salmonella* (Lozica et al., 2022; Poulsen et al., 2020). Gram-negative bacteria, including *Escherichia coli* and Salmonella, release lipopolysaccharide (LPS). This triggers a pronounced inflammatory response characterized by elevated pro-inflammatory cytokines, thereby inducing salpingitis (Song et al., 2023). To prevent and treat salpingitis and other poultry diseases, antibiotics such as amoxicillin, cephalosporins and ciprofloxacin have been widely used (Bisgaard, 1995; Lin et al., 2019). However, a withdrawal period for eggs is required during and after medication, which reduces productivity and economic returns. Moreover, the long-term use of antibiotic growth promoters (AGPs) has raised concerns regarding drug residues and antimicrobial resistance (Yang et al., 2019). Since the prohibition of AGPs in Chinese poultry production in 2020, alternatives such as Chinese herbal extracts (CHEs) have gained increasing attention for their potential in enhancing health and productivity in animal husbandry (Bozkurt et al., 2009; Dhama et al., 2015).

Scutellaria baicalensis (SB) is a traditional Chinese medicinal herb with well-documented bioactive properties. Accumulating evidence indicates that Chinese herbal extracts (CHEs) derived from SB can contribute to disease prevention and health promotion, exhibiting efficacy in the management of metabolic and inflammatory disorders such as diabetes (Zheng et al., 2021), gastric cancer (Lu et al., 2022), and ulcerative colitis(Liu et al., 2022). SB and its extracts demonstrate anti-inflammatory, antibacterial, and immunomodulatory activities(Ahmad, 2021; Feng et al., 2022; Long et al., 2021; Luo et al., 2021). For instance, oral administration of SB extract (SBE) enhanced the expression of tight junction proteins ZO-1 and occludin in a rat model of colitis(Zhu et al., 2020). In poultry, SBE alleviated Mycoplasma gallisepticum-induced lung inflammation by suppressing pro-inflammatory cytokines (TNF-α, IL-1β, IL-8) and NF-κB expression (Ishfaq et al., 2019). In cellular models, macrophages play a central role in innate immune responses to pathogens and LPS challenge, releasing key inflammatory mediators including TNF-α, IL-6, and nitric oxide synthase (NOS) (Cheon et al., 2015; Lin et al., 2014). The NF-κB signaling pathway is critically involved in regulating inflammation. Phosphorylation of IκBα and AKT can inhibit NF-κB activation and its subsequent nuclear translocation, thereby reducing the production of inflammatory cytokines (Gaskin et al., 2007; Sag et al., 2008).

However, to the best of our knowledge, the therapeutic potential and underlying mechanism of SBE specifically against salpingitis in laying hens remain unexplored. While a prior study by Ishfaq et al. (2019) demonstrated the efficacy of SBE in alleviating *Mycoplasma gallisepticum* induced lung inflammation in chickens, salpingitis presents a distinct pathological challenge localized to the reproductive tract, with significant implications for egg production (Ishfaq et al., 2019). Although SBE has demonstrated anti-inflammatory potential, its mechanism of action involving the NF-κB pathway in avian salpingitis remains unclear. Therefore, this study is the first to evaluate SBE in a laying hen model of salpingitis induced by *Escherichia coli* and *Staphylococcus aureus*, aiming to investigate its protective effects using an LPS-induced inflammatory model in HD11 macrophages *in vitro*, and a bacterial challenge model in laying hens *in vivo*, with a focus on NF-κB signaling modulation.

### Materials and methods

All procedures were approved by the Animal Care Committee of Guizhou University of Traditional Chinese Medicine (No. GUT21081112-3).

### Bacteria strains and treatments

*Escherichia coli* (ATCC 25922) and *Staphylococcus aureus* (ATCC 6538) were cultured on LB agar (Soleibao Biotechnology Co., Ltd., Beijing, China). The HD11 chicken macrophage-like cell line was obtained from Yangzhou University and cultured in RPMI1640 (Gibco®, Grand Island, NY, USA) supplemented with 10 % FBS (Gibco®). Cells were maintained at 37 °C in a humidified 5 % CO_2_ incubator (SANYO, Osaka, Japan). The scutellaria baicalensis extract (SBE) was obtained from Guizhou Pharmaceutical Group Co. Ltd. (Guiyang, Guizhou, China). Amoxicillin (purity, 40 %) was obtained from Guizhou Pharmaceutical Group Co. Ltd. (Guiyang, Guizhou, China).

Antimicrobial testing used TSA plates prepared with 12 mL molten medium. Bacterial suspensions (*E. coli* OD0.89, *S. aureus* OD0.87) were diluted 10^3^-10^6^ × in TSB. Filter discs soaked with SBE (20 μL), gentamicin (40 μg/mL positive control), or water (negative control) were placed on inoculated plates. Zones of inhibition were measured after 12-14 h incubation at 37°C.

### Animals, management and salpingitis model

A total of 180 500-day-old Hailanhe laying hens (Hubei Fuqiang Poultry Breeding Co. Ltd.) were randomly assigned to experiment groups (6 cages/group, 6 hens/cage). Hens of this age, corresponding to the later phase of the first laying cycle where egg production remains high but physiological stress and susceptibility to reproductive tract infections like salpingitis increase, were selected to establish a clinically relevant disease model. The hens were housed under controlled conditions (18±2°C, 55-65 % humidity, 12 h light/dark) with ad libitum access to diets (Table 1) and water.

**Table 1:** Basic diet composition and nutrient level.

Raw material	Content (%)	Nutrient level	Content
Corn	61.0	Metabolizable energy (MJ/kg)	13.89
Corn gluten powder	2.2	Crude protein	18.30
Soybean meal	15.5	Lysine	0.8
Rapeseed meal	2.1	Methionine	0.3
Cottonseed meal	5.0	Methionine + cystine	0.84
Beer meal	1.2	Calcium	0.9
Calcium hydrogen phosphate	1.5	Total phosphorus	0.3
Stone powder	9.0		
Salt	0.34		
L-Methionine	0.06		
Lysine	2		
Vitamin mix	0.1		
Total	100.00		

Note: Vitamin premix feed composition: VA 12500 IU, VD3 3500 IU, VE 20 IU, VK3 2.65 mg, VB1 2.00 mg, VB2 6.00 mg, VB6 3.00 mg, VB12 0.025 mg per kg diet; Trace element premix includes: biotin 0.0325 mg, folic acid 12.00 mg, pantothenic acid 50 mg, niacin 50.00 mg, copper 8 mg, iron 100 mg, zinc 40 mg, manganese 100 mg, selenium 0.16 mg, iodine 0.35 mg; The metabolizable energy is calculated and the rest is measured.

To establish a robust salpingitis model that reliably induces measurable inflammatory and pathological changes, hens received three intraperitoneal injections of *E. coli* (2.0 × 10^8^CFU/mL) and *S. aureus* (2.0 × 10^8^CFU/mL) in a ratio of 1:2. This challenge dose was selected based on preliminary studies and previous reports demonstrating consistent induction of salpingitis under controlled conditions(Liu, Yan, Li, Yu, Huang, Bai, Liu, Wang, Liu, Zhu, Xiao, Guo, Liu, Zhang, Yang, He, Zeng and Zeng, 2024; Yan, Liu, Huang, Li, Yu, Xia, Liu, Bai, Wang, Guo, Liu, Yang, Zeng and He, 2023) and it was confirmed to effectively induce salpingitis in our preliminary experiments. Although this model resulted in some mortality (*n* = 144 survivors out of 180), it ensured that the remaining birds exhibited clear signs of infection, allowing for meaningful evaluation of SBE’s therapeutic effects. We acknowledge that survivors may have varying degrees of immune activation. To mitigate this, surviving birds were randomly divided into four groups (36/group): control, amoxicillin (ANN, 100 mg/kg diet), SBE-L (100 mg/kg diet), SBE-H (300 mg/kg diet). These dietary interventions were initiated 24 h post-infection and continued for 21 days. Fallopian tube samples were collected on days 14 and 21 post-infection. For histological examination, fallopian tube tissues were fixed in 4 % paraformaldehyde, embedded in paraffin, and sectioned for hematoxylin and eosin (H&E) staining. For molecular analysis, fallopian tube tissue samples were snap-frozen in liquid nitrogen and stored at −80 °C until use. Blood samples were collected on day 21 post-infection. Plasma was subsequently separated by centrifugation at 2000 × *g* for 10 min at 4 °C and stored at −20 °C for subsequent analysis.

### Cell viability

Cell viability was assessed using the MTT assay. HD11 cells were seeded at a density of 2 × 10^4^ cells per well in a 96-well plate and treated with LPS (1 μg/mL) for 72 h. The experiment was performed with three independent biological replicates, each with technical triplicates. Following treatment, 20 μL of MTT solution (5 mg/mL in PBS) was added to each well, and the plates were incubated for 4 h at 37 °C. Subsequently, the formazan crystals formed were dissolved by adding 150 μL of dimethyl sulfoxide (DMSO) per well. The absorbance was measured at 490 nm using a Biotek Synergy 2 microplate reader. The assay was performed in accordance with the manufacturer's instructions (MTT assay kit, Langdun, Shanghai).

### Cytokines assay

LPS-treated HD11 cells (1 μg/mL) were incubated with SBE at concentrations ranging from 0 to 18 μg/mL for 24 to 72 h. The culture supernatant was then collected by centrifugation at 1000 × *g* for 10 min at 4 °C and stored at −20 °C until analysis. Supernatant from three independent biological replicates were collected, and then the levels of TNF-α, IFN-γ, IL-1β, IL-2, and IL-6 in both cell culture supernatants and plasma were measured using commercial ELISA kits (Langdun, Shanghai) according to the manufacturers’ instructions.

### RNA extraction, cDNA synthesis and qPCR

Total RNA from HD11 cells and fallopian tube tissues were isolated using 1 mL TRIZOL reagent according to the protocol. For *in vitro* studies, RNA was extracted from three independent biological replicates. RNA concentration was measured, and reverse transcribed into cDNA was performed according to the kit introductions (TaKaRa, Dalian, China). The qPCR mixtures were prepared on ice according to the manufacturer’s instructions. Subsequently, they were added to the MyiQ2 PCR system (myiQ2, Bio Rad, USA) to detect the mRNA expression. The mRNA expression was determined by qPCR using the Power SYBR Green PCR Master Mix (Invitrogen, USA) and results were analyzed by 2^-ΔΔCt^ method. The thermal cycling conditions included an initial step at 95°C for 10 min, followed by 40 cycles of 95°C for 20 s, 60°C for 20 s. The primers were designed with the Primer 5.0 software and synthesized by Sangon Biotech (Shanghai, China), as shown in Table 2.

**Table 2:** The primers sequence and parameters.

Primers	GenBank number	Primers sequence (5′→3′)	Orientation	Product length (bp)	Annealing Temperature (°C)
β-actin	L08165.1	gtgtgatggttggtatgggc	Forward	225	59
		ctctgttggctttggggttc	Reverse		
IL-1β	NM_000576	tctgtcattcgctcccacat	Forward	179	59
		agagagcacaccagtccaaa	Reverse		
IL-2	S77834.1	cctcaactcctgccacaatg	Forward	200	59
		tgtgagcatcctggtgagtt	Reverse		
IL-6	NM_000600.5	agtcctgatccagttcctgc	Forward	196	59
		ctacatttgccgaagagccc	Reverse		
IL-10	M57627.1	gttctttggggagccaacag	Forward	155	59
		gctccctggtttctcttcct	Reverse		
IL-12	AF101062.1	aatgttcccatgccttcacc	Forward	167	58
		ccaatggtaaacaggcctcc	Reverse		
IFN-γ	NM_205149.2	ccactcatacctgctcagct	Forward	153	58
		cctctcagttgcttcagtgc	Reverse		
TNF-α	NM_000594.4	gtcaacctcctctctgccat	Forward	188	59
		ccaaagtagacctgcccaga	Reverse		
NF-κB	NM_204413.2	actactcggtgactgctgac	Forward	171	59
		tgatctgctggctagggatg	Reverse		
IκBα	NM_001105720.2	gaaagctggctgtgatcctg	Forward	196	58
		catcagcacccaaagtcacc	Reverse		
AKT	AF039943.1	atgaaatgatgtgtggccgg	Forward	222	59
		gccaaacaatgccagcaaag	Reverse		

### Western blot

HD11 cells were subjected to LPS and SBE treatments as described previously. Subsequently, the cells were washed three times with 4°C PBS and collected. The cell samples were collected from three independent biological experiments. Protein samples were extracted from HD11 cells and fallopian tube tissue with RIPA buffer. Cytoplasmic proteins and nuclear proteins from HD11 cells were fractionated using a CelLytic™ NuCLEAR™ Extraction Kit (Sigma-Aldrich Co. LLC, Beijing, China). Protein concentrations were determined using a BCA Protein Assay Kit (Beyotime Institute of Biotechnology, Jiangsu, China). Proteins were separated by SDS-PAGE and blotted with the antibody of NF-κB (1:1500, ab32536, Abcam, Beijing, China), IκBα phosphorylation (1:1500, ab92700, Abcam, Beijing, China) and AKT (1:1000, ab179463, Abcam, Beijing, China). After several washes with Tris-buffered saline containing 0.1 % Tween 20, the membranes were incubated with an anti-mouse HRP-conjugated secondary antibody (1:5000 dilution, ab6829, Abcam, Beijing, China). Subsequently, the signal was detected using ECL substrate (Bio-Rad). The same membrane was incubated with β-actin (1:1000, ab6276, Abcam, Beijing, China) and lamin B (1:1000, ab133741, Abcam, Beijing, China) served as loading controls of cytoplasmic and nuclear fractions, respectively.

### Statistical analysis

Data were analyzed using one-way ANOVA followed by Tukey’s post-hoc test for multiple group comparisons. The assumptions of normality and homogeneity of variance were verified using the Shapiro-Wilk test and Levene’s test, respectively. All analyses were performed using SPSS 20.0 for Mac (IBM Corporation, Armonk, NY). The results are presented as means ± SE of the mean, and differences were considered statistically significant at *P* < 0.05.

## Results

### SBE mitigates LPS-induced cytotoxicity and inflammation in HD11 cells

**Fig. 1:**
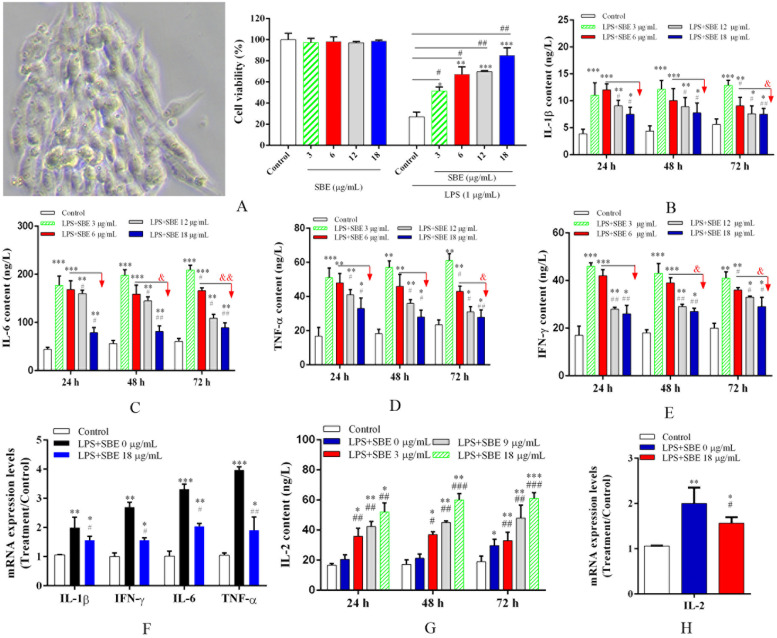
The SBE cytotoxicity and regulating inflammation in HD11 cell model. (A) Cytotoxicity; (B) Interleukin (IL)-1β; (C) IL-6; (D) Tumor necrosis factor (TNF)-α; (E) Interferon (IFN)-γ; (F) Pro-inflammatory cytokines gene expression; (G) Anti-inflammatory cytokine IL-2, (H) Anti-inflammatory cytokine gene expression. Cytotoxicity was assessed by MTT assay with SBE treatment at doses of 3, 6, 12, and 18 μg/mL for 72 h. The cytokines were performed by ELISA assay. The gene expression was measured by qPCR using the samples in the group of SBE treated with a dose of 12 μg/mL for incubating 72 h. All data are derived from three independent biological replicates (*n* = 3). Values represent the mean ± SE (bars). * Means *P* < 0.05, ** means *P* < 0.01 and *** means *P* < 0.001 compared with the control group. # Means *P* < 0.05, ## means *P* < 0.01 and ### means *P* < 0.001 compared with the only LPS treated group. & Means *P* < 0.05, && means *P* < 0.01 and &&& means *P* < 0.001 compared with the 24 h.

The cytotoxicity of SBE was examined under experimental conditions (up to 18 μg/mL and 48 h) using the MTT assay. HD11 cells were treated with various concentrations of SBE (3-18 μg/mL) for 72 h. As shown in Fig. 1A, SBE showed similar effects at doses ranging from 3 to 18 μg/mL doses, with no toxicity observed at any concentration. Therefore, SBE was not toxic to HD11 cells under these conditions.

To assess the role of SBE in regulating inflammation, we measured the levels of pro-inflammatory cytokines (IL-1β, IL-6, TNF-α and IFN-γ) and anti-inflammatory cytokines IL-2 in the LPS-induced HD11 cells supplemented with SBE. Cells were treated with LPS for 8 h, followed by four different doses of SBE (3, 6, 12, and 18 μg/mL) after 24, 48, and 72 h. As illustrated in Fig. 1, SBE treatment significantly decreased the levels of pro-inflammatory cytokines IL-1β (Fig.1B), IL-6 (Fig.1C), TNF-α (Fig.1D),and IFN-γ (Fig.1E) (*P* < 0.05). As indicated by the red arrows in Fig. 1, the total contents of IL-1β, IL-6, TNF-α and IFN-γ were decreased after SBE treatment for 48- and 72 h compared with 24 h (*P* < 0.05). The data showed that SBE alleviated the LPS induced inflammatory response in HD11 cells in a time-dependent manner. Furthermore, the gene expression levels of the pro-inflammatory cytokines IL-1β, IL-6, TNF-α, and IFN-γ were significantly up-regulated in the LPS-induced HD11 cells treated with 0 μg/mL SBE compared with the control group (*P* < 0.05), but were significantly decreased in the 18 μg/mL SBE group compared with the 0 μg/mL SBE group (Fig.1F).

Conversely, IL-2 levels were significantly elevated by SBE at doses of 0, 3, 9, and 18 μg/mL after 24, 48, and 72 h (Fig.1G), indicating a dose-dependent increase in IL-2 production. The results showed that the total content of IL-2 was significantly increased at SBE treatment for 72 h (*P* < 0.05). Additionally, IL-2 mRNA expression levels were evaluated following an 8-hour LPS pretreatment and subsequent 72 h incubation with 18 μg/mL SBE. The results showed a significant upregulation of IL-2 gene expression compared to both control and LPS-only treated groups (Fig.1H).

### SBE protect against LPS-stimulated HD11 cells by inhibiting NF-κB signaling

**Fig. 2:**
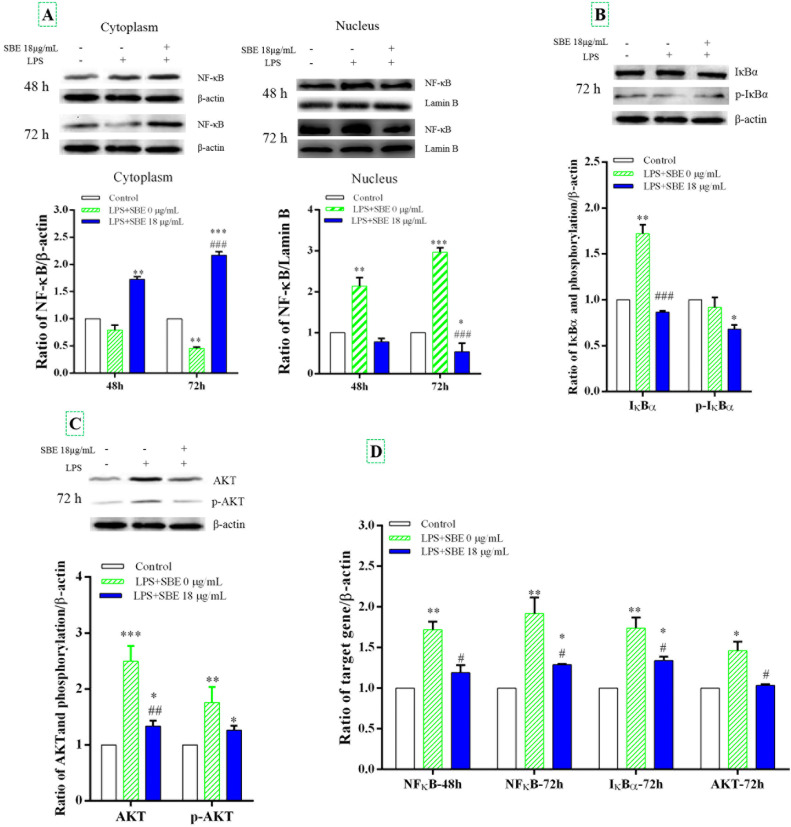
Effects of SBE on NF-κB pathway in HD11 cells after LPS treatment. The data shown are the results of NF-κB nuclear translocation (A), Quantitative data of IκBα (B), Quantitative data of AKT (C) and mRNA levels of NF-κB, IκBα and AKT (D). HD11 cells were treated with SBE at a dose of 12 μg/mL for 48- and 72 h to analyze NF-κB by western blot. The cells were treated with SBE at a dose of 12 μg/mL for 72 h to analyze protein expression of IκBα and AKT by western blot. The Western blot images are representative of three independent experiments. The mRNA levels of NF-κB, IκBα and AKT was analyzed by qPCR from three independent biological replicates (*n* = 3). β-actin and Lamin B were used as internal control for cytoplasmic and nuclear extracts, respectively. For panels B and C, the same β-actin control blot is shown. The mRNA levels of NF-κB, IκBα and AKT was analyzed by qPCR. Values represent the mean ± SE (bars). * *P* < 0.05, ** *P* < 0.01 and *** *P* < 0.001 vs. control, # *P* < 0.05, ## *P* < 0.01 and ### *P* < 0.001 vs. LPS treatment.

We examined whether SBE affects NF-κB signaling, as assessed by the nuclear translocation of NF-κB in HD11 cells. The results showed that cytosolic concentrations of NF-κB decreased in the control group at 48 h and significantly decreased at 72 h. Cytosolic concentrations of NF-κB were significantly increased in the 18 μg/mL SBE group for 48- and 72 h. The cytosolic concentrations of NF-κB at 72 h was lower than that at 48 h in the 0 μg/mL group, but the opposite was observed in the 18 μg/mL group (Fig. 2A). Furthermore, SBE significantly inhibited the translocation of NF-κB from the cytoplasm to the nucleus at 18 μg/mL for 72 h compared to the control group and 0 μg/mL SBE groups. These data clearly showed that SBE treatment significantly increased cytosolic concentrations of NF-κB while decreasing nuclear translocation of NF-κB. It is known that IκBα phosphorylation facilitates the nuclear translocation of NF-κB. However, SBE treatment significantly decreased IκBα phosphorylation at 18 μg/mL (Fig.2B). Additionally, protein levels of AKT (*P* < 0.05; Fig. 2C) in the 18 μg/mL SBE group were lower than those in the 0 μg/mL SBE group. Similarly, mRNA levels of NF-κB, IκBα, AKT was significantly suppressed expression with SBE supplementation (Fig.2D).

### SBE ameliorated salpingitis in laying hens induced by *Escherichia coli* and *Staphylococcus aureus*

No mortality occurred in any group during the experiment. Egg production in laying hens significantly decreased following infection with *Escherichia coli* and *Staphylococcus aureus*, but was significantly increased by ANN and SBE treatments (*P* < 0.05, Table 3). Blood samples collected on day 21 were used to analyze physiological indexes, including white blood cells, neutrophils, eosinophils, basophils, monocytes and lymphocytes, to evaluate the anti-inflammatory effect of SBE. The results showed that white blood cells, neutrophils, eosinophils, basophils, monocytes and lymphocytes in the SBE-L, SBE-H and ANN groups were significantly lower than those in the positive control group (*P* < 0.05). Additionally, plasma levels of the anti-inflammatory cytokines IL-2 in the SBE-L and SBE-H groups were significantly higher than those in the control group (*P* < 0.05). Interestingly, the IL-2 content in the SBE-H group was higher than those in the ANN group (*P* < 0.05). The levels of pro-inflammatory factors IFN-γ and TNF-α in SBE-L and SBE-H groups were significantly lower than those in the control group (*P* < 0.05). Based on the above results, it was indicated that SBE could relieve salpingitis in laying hens caused by bacteria by regulating the levels of inflammatory factors.

**Table 3:** Effect of dietary SBE supplementation on egg production, blood physiological indexes and inflammatory factors of laying hens.

Items	Treatments^1^				SEM^2^	P value
	Control	ANN	SBE-L	SBE-H		
Egg number (21d)	14^a^	36^b^	27^b^	32^b^	1.78	0.013
White blood cells (1 × 10^9^/L)	37.310^a^	26.330^b^	25.643^b^	25.423^b^	2.193	0.039
Neutrophils (1 × 10^9^/L)	12.095^a^	2.766^b^	3.203^b^	2.233^b^	1.871	<0.001
Eosinophils (1 × 10^9^/L)	10.297^a^	2.126^b^	3.995^b^^c^	3.768^b^^c^	1.209	<0.001
Basophils (1 × 10^9^/L)	0.120^a^	0.016^b^	0.030^b^	0.013^b^	0.042	0.028
Monocytes (1 × 10^9^/L)	12.509^a^	0.052^b^	0.058^b^	0.215^b^	0.008	<0.001
Lymphocyte (1 × 10^9^/L)	29.700^a^	21.887^b^	19.103^b^^c^	19.855^b^^c^	1.901	0.004
IL-2 (pg/ml)	9.291^a^	22.116^b^	23.035^b^	31.618^c^	1.746	<0.001
IL-10 (pg/ml)	7.40^a^	14.36^b^	12.13^b^	13.04^b^	0.13	0.032
TNF-α (pg/ml)	31.59^a^	26.63^b^	25.01^b^	29.20^b^	0.062	0.041
IFN-γ (pg/ml)	42.70^a^	26.87^b^	28.13^b^	30.85^b^	3.06	0.023

a,b,c Means with different superscripts in each row differ significantly (*P* < 0.05). Abbreviations: ANN, amoxicillin; SBE, scutellaria baicalensis extract;.

1 Control, salpingitis laying hens treated with an antibiotic-free diet; ANN, amoxicillin group, 100 mg/kg via diet supplementation after injection a mixture of *Escherichia coli* and *Staphylococcus aureus* suspension; SBE-L, low-dose SBE group (SBE-L), 100 mg/kg via diet supplementation after injection a mixture of *Escherichia coli* and *Staphylococcus aureus* suspension; SBE-H, high-dose SBE group (SBE-H), 300 mg/kg via diet supplementation after injection a mixture of *Escherichia coli* and *Staphylococcus aureus* suspension. Each treatment group consisted of 36 hens (*n* = 36).

2 SEM: standard error of the means.

**Fig. 3:**
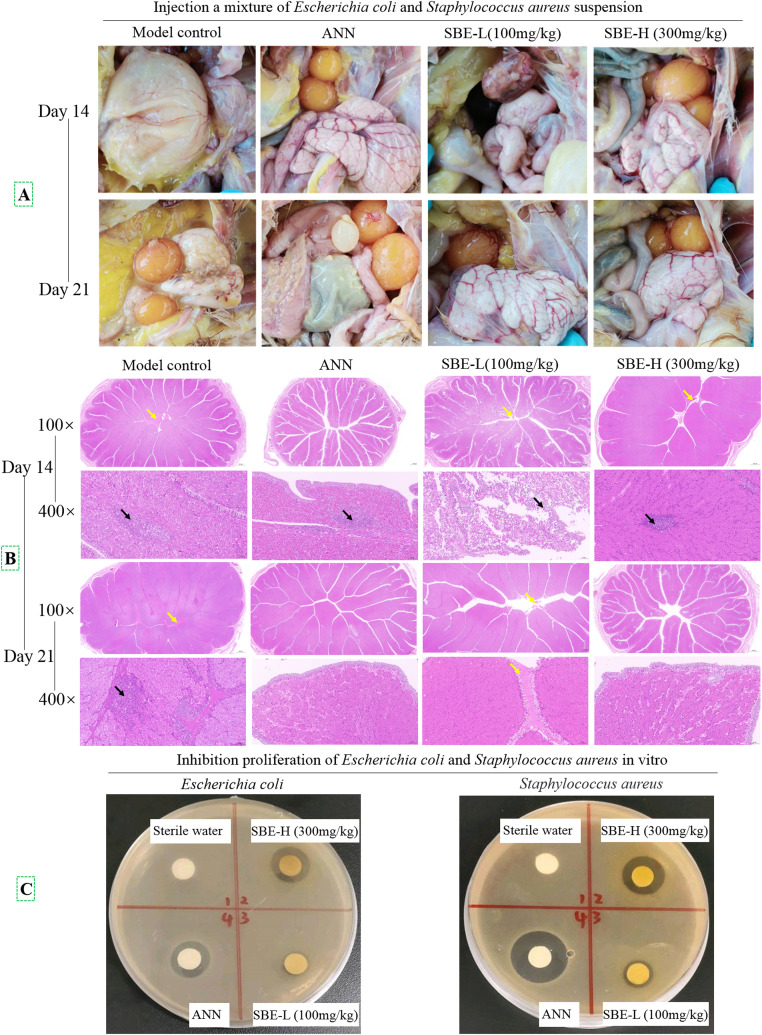
Protective effects of SBE on the pathological changes of laying hens salpingitis induced by *Escherichia coli* and *Staphylococcus aureus* infection. (A) Anatomic assessment; (B) Histopathological assessment of HE staining on day 14 and 21; (C) Bacteriostatic zone test.

Anatomic assessment was performed to detect the formation and severity of lesions in the fallopian tubes of different groups (Fig.3A). In the model group, egg yolk lesion (day 14) or rupture (day 21) were observed in the abdominal cavity, which also exhibited an unpleasant odor. No obvious inflammatory changes were observed in the fallopian tubes of chickens in the groups of ANN and 300mg/kg SBE treatment group (days 14 and 21). However, the 100 mg/kg SBE group exhibited hyperemia and caseous inflammatory secretions. A histopathological assessment was performed to detect the presence and severity of lesions in the different groups of fallopian tubes (Fig.3B). Laying hens with salpingitis revealed typical lesions, including eosinophilic substances in the oviduct (yellow arrow) and lymphocyte infiltration (black arrow). In contrast, the tissue damage in the SBE groups was significantly reduced compared with the model group. The SBE could inhibit the proliferation of *Escherichia coli and Staphylococcus aureus* by the bacteriostatic zone test (Fig.3C).

### Regulation of NF-κB signalING in *Escherichia coli* and *Staphylococcus aureus*-induced laying hens salpingitis model

**Fig. 4:**
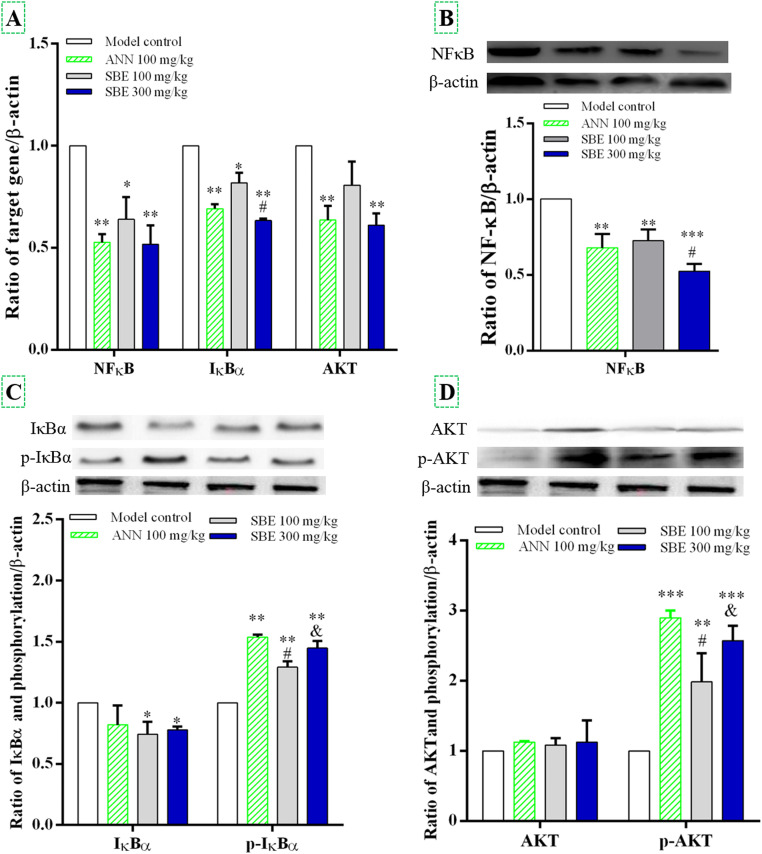
Effects of SBE on regulating NF-κB signal to alleviate *Escherichia coli* and *Staphylococcus aureus* induced salpingitis of laying hens. (A) NF-κB, IκBα and AKT mRNA expression; (B) NF-κB protein expression and quantitative data; (C) IκBα protein expression and quantitative data; (D) AKT protein expression and quantitative data. The NF-κB, IκBα and AKT mRNA expression and protein expression in the fallopian tube tissues from laying hens with the 100 mg/kg and 300 mg/kg SBE supplementation was assayed by RT-PCR and western blot, respectively. β-actin was used as internal control. Values represent the mean ± SE (bars). * *P* < 0.05, ** *P* < 0.01 and *** *P* < 0.001 vs. model control, # means *P* < 0.05 vs. ANN group. & means *P* < 0.05 vs. SBE-L group.

To determine whether the effect of SBE on improving *Escherichia coli* and *Staphylococcus aureus* induced salpingitis in laying hens is associated with NF-κB signaling pathways, the expression of NF-κB, IκBα and AKT mRNAs and proteins in fallopian tube tissues was examined (Fig. 4). The mRNA levels of NF-κB, IκBα and AKT were significantly suppressed in the ANN and SBE groups compared to the model group (*P* < 0.05), except for AKT mRNA in the 100 mg/kg SBE group (Fig. 4A). Among these, IκBα mRNA was obviously increased in the 300 mg/kg SBE group compared to the ANN group (*P* < 0.05). Total protein expression of NF-κB was significantly decreased in the ANN and SBE groups compared to the model control (*P* < 0.05, Fig.4B). Phosphorylation levels of IκBα and AKT protein were significantly down-regulated in fallopian tube tissues with ANN and SBE treatment compared to the model control group (*P* < 0.05, Fig. 4C and Fig. 4D). Phosphorylation levels of IκBα in the ANN and SBE 300 mg/kg groups were higher than those in the 100 mg/kg SBE group (*P* < 0.05). The results of AKT protein phosphorylation level was similar with the IκBα protein. These results indicated that SBE supplementation could relieve salpingitis in laying hens by inhibiting the NF-κB signaling associated with IκBα and AKT protein expression.

## Discussion

The present study has established that SBE exerts anti-inflammatory effects in both LPS-stimulated HD11 cells and in a laying hen model of salpingitis. Our findings are consistent with those from recent studies on other plant extracts in poultry. For instance, Tran et al. (2023) reported that Prinsepiae Nux extract reduced pro-inflammatory cytokines (IL-6 and IL-1β) in broilers, supporting the role of plant extracts in modulating inflammatory responses (Tran et al., 2023). Similarly, Thakur et al. (2024) showed that *Artemisia nilagirica* leaf extract protected broilers from *E. coli* challenge by reducing inflammation and tissue damage (Thakur et al., 2024). These studies, along with our results, highlight the potential of plant extracts as anti-inflammatory agents in poultry. It is well established that IL-1β, IL-6, and TNF-α play pivotal roles in mediating inflammatory responses (Marafini et al., 2019). Our findings align with previous reports indicating that CHEs can mitigate inflammatory damage by modulating cytokine profiles, notably by reducing levels of pro-inflammatory mediators in various animal models (Liu et al., 2020; Sandoval-Ramírez et al., 2021; Wang et al., 2018; Zhao et al., 2020). It has been reported that LPS-induced inflammatory responses could elevate NF-κB levels, which in turn regulate the expression of numerous genes involved in inflammation (Brasier, 2006; Lai et al., 2009; Lu et al., 2017, 2008). Liu et al. (2020) demonstrated that Camellia sinensis extract ameliorated intestinal inflammation by modulating gut microbiota and immune responses (Liu, Wang, Chen, Luo, Ma, Xiao and Zeng, 2020). Similarly, Luo et al. (2021) reported that *Astragalus* polysaccharide reduced intestinal inflammatory damage in goslings (Luo, Yang, Liu, Li, Wang, Ge and Zhang, 2021). These studies support the broad applicability of plant extracts in managing inflammatory conditions in poultry. In the current study, we further elucidated the mechanism by which SBE inhibits NF-κB nuclear translocation via the AKT/IκBα pathway. This aligns with the work by Huang et al. (2012) and others who have shown that natural compounds can suppress NF-κB signaling to attenuate inflammation (Gilmore, 2006; Huang et al., 2012).

For the *in vivo* study, a salpingitis model was established in laying hens using *Escherichia coli* and *Staphylococcus aureus* infection. As expected, infected birds exhibited elevated levels of IFN-γ and TNF-α, consistent with previous findings (Li, Xu, Liu, Liu, Dong, Zhang, Guo and Ding, 2023). These bacteria are recognized as common pathogens in avian salpingitis, often leading to pronounced inflammatory cytokine secretion and tissue damage in the oviduct (Poulsen, Kudirkiene, Jorgensen, Djordjevic, Cummins, Christensen, Christensen, Bisgaard and Thofner, 2020; Song, Li, Chen, Feng, Duan, Cheng, Chen, Wang and Min, 2023). It should be noted that the bacterial challenge used in this study was severe, leading to partial mortality, which may have selected for birds with stronger innate immunity. Nonetheless, the consistent improvements in egg production, histopathology, and inflammatory markers across SBE-treated groups support the efficacy of SBE even under such stringent conditions. Baicalin, a primary active component of SBE, exhibits a spectrum of pharmacological effects, including hepatoprotective, antitumor, antibacterial, anti-inflammatory, and antidepressant activities(Hu et al., 2021). In a colitis rat model, oral administration of baicalin (100 mg/kg) could enhance mRNA expression levels of ZO-1 and occludin, ameliorating TNBS-induced colitis (Zhu, Xu, Zhao, Shen, Shen and Zhan, 2020). In addition, SBE has been proven to alleviate *Mycoplasma gallisepticum* induced inflammatory damage in the lung by increasing commensal bacterium Bacteroides fragilis and modulating phenylalanine metabolism (Ishfaq, 2019). Our results further indicate that SBE supplementation enhances anti-inflammatory capacity in laying hens challenged with bacterial infection.

To elucidate the underlying mechanism, we examined the NF-κB signaling pathway. Inhibition of NF-κB activation has been identified as a critical strategy for reducing bacteria-induced cytokine production (Brasier, 2006; Gilmore, 2006; Mann et al., 2017; Perkins, 2007), particularly for pro-inflammatory cytokines such as IL-1β, IL-6, TNF-α, and IFN-γ (Chang et al., 2011a, 2011b). NF-κB is sequestered in the cytoplasm by inhibitory proteins, IκBs, which mask its nuclear localization signals (Huxford et al., 1998; Jacobs and Harrison, 1998). Upon activation, IκB degradation enables NF-κB nuclear translocation and subsequent cytokine gene transcription (Kearns et al., 2006; Nelson et al., 2004). Our in vitro results showed that SBE treatment significantly reduced NF-κB translocation. In vivo, SBE administration downregulated total NF-κB expression and restored phosphorylation levels of IκBα and AKT, which are essential for regulating NF-κB activity. These molecular changes correlated with a reduction in pro-inflammatory cytokines and an increase in anti-inflammatory mediators, supporting the role of SBE in mitigating inflammation through the AKT/IκBα/NF-κB axis.

Furthermore, we demonstrated that the pro-inflammatory cytokines genes were up-regulated in the LPS-stimulated HD11 cell. The SBE treatment was significantly inhibited LPS-induced pro-inflammatory cytokine gene expression. This suggests that anti-inflammatory cytokines are involved in the SBE-mediated inhibition of LPS-induced NF-κB activation in HD11 cells. Additionally, the total protein expression level of NF-κB was significantly decreased in the SBE treatment compared with the laying hens salpingitis model group (Fig. 4B). Phosphorylation levels of IκBα and AKT protein were decreased in the salpingitis model group, but SBE treatment suppressed this effect. On one hand, SBE treatment promoted the phosphorylation of AKT, which led to reduced NF-κB activation, thereby inhibiting the production of inflammatory factors such as IL-1β, IL-6, and TNF-α. On the other hand, SBE treatment can increase anti-inflammatory factors content to relieve inflammation(Lee et al., 2017). Thus, blocking the NF-κB signaling pathway can mitigate inflammation(Yan et al., 2018). These effects are also reflected in the results of egg production (Table 3) and the anatomic and histopathological assessment (Fig.3). These results showed that SBE markedly increased the number of eggs produced, decreased eosinophilic substances (yellow arrow), and reduced lymphocyte infiltration (black arrow) in the tubal lumen of laying hens with salpingitis. In general, bacteria infection or LPS challenge increase the activity of the NF-κB signaling pathway in the fallopian tubes of laying hens and in HD11 cells. More importantly, SBE obviously inhibited the activation of the NF-κB signaling pathway, thereby relieving salpingitis in laying hens.

However, research on the prevention and treatment of bacterial diseases by SBE is still at the stage of laboratory analysis and experimental animal evaluation. Subsequently, we plan to apply it to animal feeding. In addition, the target animals used are relatively few, and to date, only laying hens have shown a relatively good therapeutic effect. Future plans include verification in weaned-piglets with bacterial diarrhea and other conditions. So far, the results using feed supplementation have been satisfactory, and we will consider clinical translation pathway in future studies.

## Conclusion

In summary, this study elucidates the molecular mechanism by which SBE exerts anti-inflammatory effects in cellular and avian salpingitis models. We demonstrated that SBE attenuates the inflammatory response by inhibiting NF-κB nuclear translocation through modulation of the AKT/IκBα pathway. This inhibition resulted in decreased pro-inflammatory cytokine production and enhanced anti-inflammatory activity. These beneficial effects were associated with improved health and productivity in laying hens, supporting the potential of SBE as an effective antibiotic-free alternative for managing bacterial salpingitis in poultry production.

## Ethical approval and consent to participate

The animal experiments were approved by the Animal Welfare and Research Ethics Committee at Guizhou University of Traditional Chinese Medicine (Guiyang, China). All protocols and studies involving animals were conducted following the guiding principles of the Animal Care and Use Committee of Guizhou University of Traditional Chinese Medicine (Guiyang, China; No. GUT21081112-3)

## Availability of data and materials

All data and materials are available. The data that support the findings of this study are available from the corresponding author upon reasonable request.

## Funding

The Science Technology Support Plan of Guizhou Province (Qian Science and technology cooperation Support No. [2020]1Y045 and No.[2022]131), the Innovation and Entrepreneurship Training Program of Guizhou Province (202110662054), Health Commission of Guizhou Province (gzwkj2021-535), Scientific Research Foundation for Young and Middle-aged teachers of Fujian Province (JAT200997) and PhD Launch Fund (Your Traditional Chinese Medicine Doctor Start-up [2019] No.144).

## CRediT authorship contribution statement

**Jing Zhang:** Writing – review & editing, Writing – original draft, Validation, Supervision, Software, Resources, Project administration, Methodology, Investigation, Funding acquisition, Formal analysis, Data curation. **Qianmei Zhang:** Writing – review & editing, Writing – original draft, Supervision, Software, Resources, Methodology, Investigation, Formal analysis, Data curation. **Shiyong Wang:** Writing – review & editing, Writing – original draft, Software, Resources, Methodology, Investigation, Formal analysis, Data curation. **Yuechun Yang:** Writing – review & editing, Investigation, Funding acquisition, Formal analysis, Data curation. **Lingyan Zhang:** Writing – review & editing, Methodology, Investigation, Formal analysis, Data curation. **Lihong Chen:** Writing – review & editing, Investigation, Formal analysis, Data curation. **Zhenglu Xie:** Writing – review & editing, Writing – original draft, Visualization, Validation, Supervision, Software, Resources, Project administration, Methodology, Investigation, Funding acquisition, Formal analysis, Data curation, Conceptualization.

## Disclosures

The authors declared that they have no conflicts of interest to this work.
